# HIV care among patients with presumptive tuberculosis in Masvingo district of Zimbabwe, 2017: how well are we doing?

**DOI:** 10.11604/pamj.2019.33.158.15847

**Published:** 2019-07-03

**Authors:** Chamunorwa Ndudzo, Jaya Prasad Tripathy, Fortune Tauro, Clemence Sibanda, Maikoro Chiramba, Amadeus Shamu, Kudzai Masinire, Tafadzwa Muchengwa, Ajay MV Kumar

**Affiliations:** 1 AIDS and TB Programme, Masvingo Province, Zimbabwe; 2 The Union South-East Asia Office, International Union Against Tuberculosis and Lung Disease, New Delhi, India; 3 International Union Against Tuberculosis and Lung Disease, Paris, France; 4 Yenepoya Medical College, Yenepoya (Deemed to be University), Mangaluru, India

**Keywords:** TB suspect, presumptive TB patients, HIV care cascade, operational research, provider initiated HIV testing, PITC, SORT IT

## Abstract

**Introduction:**

While HIV care among tuberculosis (TB) patients is successfully implemented and monitored, it is not routinely reported among “presumptive TB patients without TB”. The present study describes the ascertainment of HIV status and receipt of antiretroviral therapy (ART) and the associated factors among presumptive TB patients (with and without TB) in 35 public health facilities of Masvingo district of Zimbabwe from January to June 2017.

**Methods:**

This was an analysis of secondary programme data. We performed log binomial regression to calculate adjusted relative risks (aRR) and 95% confidence intervals (CI).

**Results:**

Of 1369 presumptive TB patients, 1181 (86%) were ascertained for HIV status (98% among those subsequently diagnosed with TB, 83% among non-TB). Of them, 748 (63%) were HIV positive, more among TB patients (69%) than those without TB (61%). Among HIV-positive patients, 475 (64%) received ART, significantly higher among TB patients (78%) compared to those without TB (57%). Patients without TB were significantly more likely to have non-ascertained for HIV status (aRR=2.4, 95% CI=1.4-5.0) and not receiving ART (aRR=1.8, 95% CI=1.6-2.0), compared to those with TB.

**Conclusion:**

We found high rates of HIV status ascertainment among presumptive TB patients. But, ART uptake was poor among “presumptive TB patients without TB”, despite implementation of “test and treat” strategy in Zimbabwe. The programme should step up the monitoring of HIV status and ART receipt among presumptive TB patients, by introducing an indicator in the quarterly reports of the national TB programme.

## Introduction

Despite great progress in the response to Tuberculosis-Human Immunodeficiency Virus (TB/HIV) syndemic globally, the morbidity and mortality remains high. According to the World Health Organization (WHO) Global TB Report 2017, an estimated 1.0 million people globally had HIV-associated TB, and 374, 000 died from it in 2016 [1]. Because HIV is treatable with highly effective antiretroviral therapy (ART) and most of the TB is curable, such high mortality is unacceptable. The WHO and the Joint United Nations Programme on HIV/AIDS (UNAIDS) have both embraced a new vision to end epidemics of HIV and TB, in line with the sustainable development goals by 2030 [2]. To achieve this, the WHO's Global TB Programme has formulated an 'End TB strategy' with an overall vision of a TB-free world (zero deaths, zero disease and zero suffering due to TB) and a goal of ending the TB epidemic (annual incidence of TB to 10 new cases or less per 100,000 population) [3]. Similarly, UNAIDS has provisionally defined ending the AIDS epidemic as reducing the number of new HIV infections and AIDS-related deaths by 90% compared to 2010 levels [2]. Both the TB and HIV worlds have set themselves ambitious 90-90-90 targets to achieve by 2020. With respect to HIV, it means diagnosing 90% of estimated PLHIV, treating 90% of those diagnosed with HIV and achieving viral suppression in 90% of those treated with ART [2]. In the case of TB, this means diagnosing and treating 90% of all people with TB, including 90% of the key populations at risk of TB and achieving 90% treatment success for all people diagnosed with TB [4]. In 2012, WHO launched its updated policy on collaborative TB/HIV activities, which recommended HIV testing for not only patients diagnosed with TB, but also to all those being investigated for possible TB (hereafter referred to as “presumptive TB”). This recommendation was based on high HIV prevalence among patients with presumptive TB [5-12] and high mortality among HIV-infected presumptive TB patients without active TB [13]. The national TB and HIV programmes in Zimbabwe took a policy decision to implement HIV testing and counselling among patients with presumptive TB before WHO launched the guidelines in 2012 [14]. While HIV care among active TB patients is widely implemented, monitored and reported, HIV testing in persons with presumptive TB is not routinely reported [15]. A previous study conducted in a single health facility in Bulawayo, Zimbabwe during 2009-13 by Dlodlo *et al*. found poor ART uptake among HIV patients without TB (~40%) compared to those who had TB (90%) [16]. Since that study, several programmatic interventions have been implemented by the national HIV programme in Zimbabwe including decentralized availability of HIV testing and ART services at all health facilities and the adoption of “test and treat” policy, meaning all PLHIV be started on ART irrespective of CD4 count or clinical staging [17]. However, whether these measures have resulted in improved HIV care among presumptive TB patients is not known. Thus, the present study was carried out to describe the ascertainment of HIV status and uptake of ART services and the associated factors among presumptive TB patients (with and without TB) attending the public health facilities of Masvingo district, Zimbabwe during January-June 2017.

## Methods

**Study design:** this was an analysis of secondary data collected routinely by the National AIDS and TB programme in Zimbabwe.

### Setting

**General setting:** Zimbabwe is a country situated in Southern Africa with a population of ~13 million [18]. Zimbabwe has 10 provinces of which eight are rural and two are city provinces. The country's Gross Domestic Product (GDP) is 113.9 billion US dollars which places it in the group of low income countries [19]. Masvingo Province is one of the country's eight rural provinces, located in the southern part of the country and has a population of 1.5 million. The province has seven districts out of which Masvingo is the study district [2]. Masvingo is predominantly a rural district with a population of 0.3 million.

**Zimbabwe NTP:** all patients presenting at health facilities are screened for signs and symptoms of TB using the TB screening questionnaire. Anyone who has cough for more than two weeks, fever, weight loss, night sweats or household contact of active TB is considered to be “presumptive TB patient” and recorded in a “Presumptive TB Register” which is placed at each health facility. Then s/he is evaluated for TB in accordance with the standard TB diagnostic algorithm using sputum smear microscopy and/or Xpert MTB/RIF assay [20]. Of the health facilities in the district, five have TB diagnostic facilities-all of these have sputum microscopy while two of these have Xpert MTB/RIF. Those who are negative bacteriologically are assessed clinically using chest radiography and other appropriate investigations and a diagnosis of clinical TB is made by the treating physicians [21]. All diagnosed TB patients are treated free of charge with daily DOTS regimen under direct supervision as per national guidelines [20]. TB treatment is available at all health facilities [20]. As part of the TB-HIV collaborative activities, HIV status is routinely ascertained among presumptive TB patients. For patients who know their status, HIV test result is documented in presumptive TB register. Patients with unknown HIV status are offered HIV testing and those found to be HIV-positive are initiated on ART free of cost [22]. HIV testing and ART services are available at all health facilities of the study district.

**Study population:** all presumptive TB patients registered in 35 health facilities of Masvingo district from 1^st^January to 30^th^ June 2017 were included in the study.

**Data collection:** the principal investigator along with a trained data collector visited all the public health facilities in the district and collected data using a structured proforma which included variables like age, sex, point of entry into care, HIV status, TB status and ART status. A master list of presumptive TB patients registered during the period January-June 2017 was prepared from the presumptive TB register. Presumptive TB registers at all entry points in the health facility were collected. The laboratory register was cross-checked to complete the list. If the patient was found in the laboratory register but not in the presumptive TB register, then he/she was included in the study population. The TB status of each patient was assessed by reviewing the sputum microscopy and Xpert MTB/RIF registers. A person whose sputum smear was positive and/or whose Xpert MTB/RIF was positive for *Mycobacterium Tuberculosis* (MTB) was considered as “bacteriologically confirmed TB”. The treatment register was cross-checked to include all those who were diagnosed as “clinical TB” and started on treatment. Name, age and sex were used as tracking variables across the registers. HIV test result was captured from the presumptive TB register. Finally, ART register was reviewed to assess the ART status of HIV-positive people.

**Data analysis and statistics:** double data entry, validation and analysis were performed using EpiData software (Version 3.1 for entry and 2.2.2.186 for analysis; EpiData Association, Odense, Denmark). Proportions were used to summarise the categorical variables. There were two key outcomes: i) ascertainment of HIV status ii) ART receipt and these are defined below.

### Operational definitions

**HIV status ascertained:** presumptive TB patients who know their HIV status (includes both with previously known HIV status and newly tested for HIV). 'Known HIV status' means patients with a documented HIV-positive result or those with a HIV-negative result in the past three months.

**Receiving ART:** this includes HIV-infected presumptive TB patients who were receiving ART during the study period (includes those previously on ART and those newly started on ART). The key predictor variables included age, gender, entry point and type of TB. Chi-square test was used to study the association of demographic and clinical variables with non-ascertainment for HIV and not receiving ART [17]. The strength of association was expressed using relative risk (RR) and adjusted RR with 95% confidence intervals (CI) using a log binomial regression model. Factors with a p value <0.2 in the unadjusted analysis were included in the regression model using STATA version 12.

**Ethics:** permission to access and use programme data was obtained from the National AIDS and TB Unit, Ministry of Health and Child Care, Zimbabwe. Ethics approval was obtained from the National Medical Research Council of Zimbabwe and The Union Ethics Advisory Group, Paris, France. Since the study involved review of existing records with no direct interaction with the patients, the need for individual informed consent was waived by the ethics committees.

## Results

There were a total of 1426 presumptive TB patients during the study period. Of them, 57 (4%) from the Zimbabwe Prison Service, Mutimurefu were excluded due to missing data. Of the remaining 1369 included in the analysis, more than half of the patients were adults in the age group of 15-44 years (780, 57%), females (690, 51%), belonged to the rural areas (842, 63%) and were referred from general outpatient department (710, 52%). Nearly a quarter of the presumptive TB patients were diagnosed with TB (330, 24%). Of them, 128 (9%) had bacteriologically confirmed pulmonary TB, 174 (13%) were diagnosed as “clinically confirmed pulmonary TB” and 28 (2%) had extra-pulmonary TB (Table 1).

**Table 1:** Demographic and clinical characteristics of presumptive TB patients registered in Masvingo district, Zimbabwe, January-June 2017

Characteristics	N	%
Total	1369	100
**Age group**		
0-14 years	84	(6)
15-44 years	780	(57)
45 years and above	505	(37)
**Gender**		
Male	661	(48)
Female	690	(51)
Not recorded	18	(1)
**Place of residence**		
Rural	842	(63)
Urban	498	(35)
Not recorded	29	(2)
**Entry point**		
General OPD	710	(52)
Laboratory	188	(14)
TB clinic	224	(16)
OI Clinic	213	(16)
Medical wards	34	(2)
**Type of TB**		
Bacteriologically confirmed pulmonary TB	128	(9)
Clinically confirmed pulmonary TB	174	(13)
Extra-pulmonary TB	28	(2)
No TB	1039	(76)

OPD=Outpatient Department; OI: Opportunistic Infection; TB=Tuberculosis

**Figure 1:**
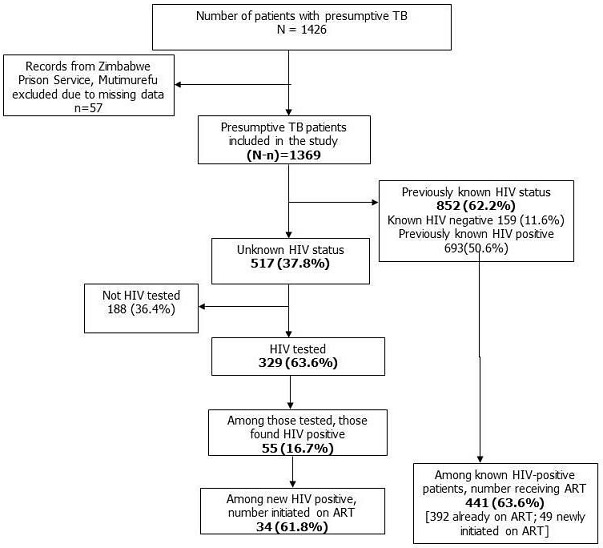
HIV status and ART uptake among presumptive TB patients registered during January-June 2017 in public health facilities of Masvingo district, Zimbabwe

**Figure 2:**
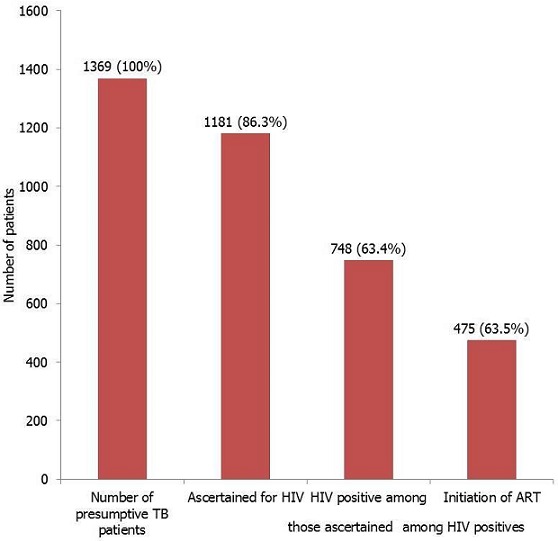
Cascade of HIV care among presumptive TB patients registered during January-June 2017 in public health facilities of Masvingo district, Zimbabwe

**Figure 3:**
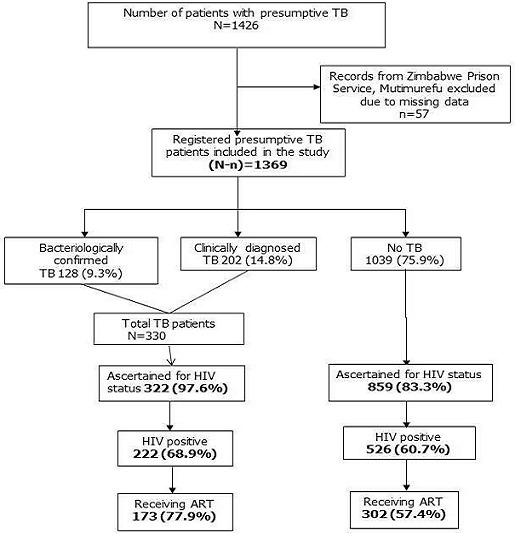
HIV status ascertainment and ART uptake among presumptive TB patients (with and without TB) registered during January-June 2017 in public health facilities of Masvingo

**HIV status and ART initiation:** of 1369 presumptive TB patients, 852 (62.2%) had prior knowledge of their HIV status (693 being known HIV positives and 159 were known HIV negatives). Out of 693 known HIV patients, 392 (57%) were already on ART and 49 (7%) were initiated on ART. Among those with unknown HIV status (n=517), 329 (63.6%) were newly tested, of which, 55 (16.7%) were found positive for HIV and of them, 34 (61.8%) were initiated on ART (Figure 1). Thus, overall, 1181 (86.3%) were ascertained for HIV status (both new and previously known) of which 748 (63.4%) were HIV positive. Among HIV-positive patients, 475 (63.5%) received ART (Figure 2). Out of 330 TB patients, 322 (97.6%) were ascertained for HIV status and 222 (68.9%) were HIV-positive. Of the latter, 173 (77.9%) were initiated on ART. Among 'presumptive TB patients without TB' (n=1039), 859 (83.3%) were ascertained for HIV status and 526 (60.7%) were HIV positive. Of them, 302 (57.4%) received ART (Figure 3).

**Factors associated with non-ascertainment of HIV status:** adjusted analysis showed that patients whose entry into care was outpatient department (OPD) or medical wards (aRR=3.7, 95% CI=1.7-8.4) and TB laboratory (aRR=8.0, 95% CI=3.5-17.8), those belonging to the age group 0-14 years (aRR=2.1, 95% CI=1.5-2.8) and those without TB (aRR=2.4, 95% CI=1.4-5.0) were significantly more likely to be non-ascertained for HIV status (Table 2).

**Table 2:** Demographic and clinical factors associated with non-ascertainment for HIV status among presumptive TB patients in Masvingo district, Zimbabwe, January-June 2017

Characteristics	Total N	Not ascertained for HIV status n (%)	Unadjusted Relative risk (95% CI)	p-value	Adjusted Relativerisk (95% CI)	p-value
**Total**	1369	188 (13.7)				
**Age group**						
0-14 years	84	28 (33.3)	2.7 (1.9-3.9)	<0.001	2.1 (1.5-2.8)	<0.001
15-44 years	780	96 (12.3)	1.0		1.0	
45 years and above	505	64 (12.7)	1.03 (0.8-1.4)	0.85	1.0 (0.7-1.2)	0.35
**Gender**						
Female	690	92 (13.3)	0.97 (0.7-1.3)	0.82	Not included	
Male	661	91 (13.8)	1.0			
**Residence**						
Rural	842	109 (12.9)	0.96 (0.7-1.3)	0.79	Not included	
Urban	498	67 (13.5)	1.0			
**Entry point**						
General OPD/wards	744	79 (10.6)	5.8 (2.8-11.9)	<0.001	3.7 (1.7-8.4)	0.001
TB Laboratory	188	101 (53.7)	29.3 (14.6-59.0)	<0.001	8.0 (3.5-17.8)	<0.001
TB/OI Clinic	437	8 (1.8)	1.0		1.0	
**Type of TB**						
No TB	1039	180 (17.3)	7.1 (3.6-14.3)	<0.001	2.4 (1.4-5.0)	0.001
TB	330	8 (2.4)	1.0		1.0	

OPD=Outpatient Department; CI=Confidence Interval; TB=Tuberculosis; OI=Opportunistic Clinic; HIV=Human Immunodeficiency Virus

**Factors associated with non-receipt of ART:** patients referred from TB/Opportunistic Infection (OI) clinic (aRR=1.4, 95% CI=1.2-1.8), TB laboratory (aRR=2.8, 95% CI=1.9-3.8) and those without TB (aRR=1.8, 95% CI=1.6-2.0) were significantly more likely to be not receiving ART (Table 3).

**Table 3:** Demographic and clinical factors associated with not receiving ART among HIV-infected presumptive TB patients registered in Masvingo district, Zimbabwe, January-June 2017

Characteristics	Total N	Not receiving ART n (%)	Unadjusted Relative risk (95% CI)	p-value	Adjusted Relative Risk (95% CI)	p-value
**Total**	748	273 (36.5)				
**Age group**						
0-14 years	23	8 (34.8)	1.01(0.57-1.79)	0.98	Not included	
15-44 years	470	162 (34.5)	1.0			
45 years and above	255	103 (40.4)	1.17(0.96-1.42)	0.11		
**Gender**						
Female	387	136 (35.1)	0.95 (0.78-1.15)	0.58	Not included	
Male	353	131 (37.1)	1.0			
**Residence**						
Rural	414	146 (35.3)	0.93 (0.77-1.12)	0.43	Not included	
Urban	323	123 (38.1)	1.0			
**Entry point**						
General OPD/wards	359	70 (19.5)	1.0		1.0	
TB Laboratory	58	56 (96.6)	4.95 (3.99-6.14)	<0.001	2.8 (1.9-3.8)	<0.001
TB/OI Clinic	331	147 (44.4)	2.28 (1.79-2.90)	<0.001	1.4 (1.2-1.8)	0.001
**Type of TB**						
No TB	526	224 (42.6)	1.93 (1.48-2.52)	<0.001	1.8(1.6-2.0)	<0.001
TB	227	49 (22.1)	1.0		1.0	

OPD=Outpatient Department; CI=Confidence Interval; TB=Tuberculosis; OI=Opportunistic Clinic; HIV=Human Immunodeficiency Virus; TB=Tuberculosis; ART=Antiretroviral therapy

## Discussion

This is the first district-wide study in Zimbabwe analysing the cascade of HIV care among presumptive TB patients after the implementation of the 'test and treat' approach. The key findings of the study are: a) high rates of HIV status ascertainment among presumptive TB patients, which was better among TB patients compared to non-TB, b) two-thirds of those ascertained for HIV status were HIV positive, again marginally higher among TB patients c) poor ART uptake, worse among HIV patients without TB compared to HIV-TB co-infected patients. The study had a few strengths. First, it was a district-wide study involving a large number of presumptive TB patients in a routine programme setting, thus reflecting the ground realities. Second, we adhered to the Strengthening the Reporting of Observational studies in Epidemiology (STROBE) guidelines to report the study findings [23]. Third, data were abstracted from multiple registers to collect information on all types of TB including clinical/extra-pulmonary TB and thus the assessment of TB diagnosis was more complete compared to previous studies on the same topic [16]. Fourth, double data entry and validation was done to minimize data entry errors. There were a few limitations. Firstly, routine programme data were used in this study which was found to be incomplete in some places. We assumed that HIV-positive presumptive TB patients whose ART status was “not recorded” as having not initiated on ART. It is possible that the patients received ART at the clinic without getting recorded in the register (less likely in our view) or were initiated elsewhere (especially outside the district) without the information being communicated to the clinic. We also assumed that presumptive TB patients whose HIV status was “not recorded” as not tested for HIV. Secondly, this study was conducted in a single district and the results may not be generalizable to the entire country. Thirdly, we did not investigate the reasons for poor ART uptake among HIV patients without TB. This needs further investigation. Fourthly, we did not capture information on the uptake of Isoniazid Preventive Therapy (IPT) among HIV patients without TB. Previous studies in Asia and Africa reporting on the diagnosis and management of presumptive TB patients have found HIV testing uptake to range between 79%-92%, similar to the findings of the present study, [5, 6, 8, 9, 12, 16] whereas other studies have reported lower uptake rates between 45-59% [10, 24]. In Zimbabwe, the high level of HIV ascertainment could be attributed to the following reasons: i) implementation of the integrated TB-HIV services wherein presumptive TB patients are offered both TB and HIV testing simultaneously, ii) deployment of full-time primary care counsellors who provide HIV counselling and testing services at all high volume health facilities, iii) decentralized availability of rapid HIV test kits in all public health facilities, at no cost to the patient [25].

High rates of HIV positivity were also observed in other studies from sub-Saharan Africa [16, 26, 27]. HIV positivity was more than 60% in both TB and non-TB groups which provides additional justification for the policy of HIV testing among presumptive TB patients. One key finding of the study was poor ART uptake especially in the non-TB group. Compared to an earlier study from Zimbabwe in 2009-13, there has been a modest improvement in ART uptake from 38% to 57% [16]. This improvement could be ascribed to the policy of 'test and treat' and the decentralised ART services in the country. However, it is well below the global targets. The time period for checking the registers for ART initiation did not extend beyond the study period which might have led to an underestimation of ART uptake. However, the effect of this limitation is likely to be minimal as this affects only those who were newly diagnosed with HIV and not started on treatment. Patients whose point of entry was OPD/wards and TB laboratories were less likely to be ascertained for HIV. The exact reasons for this are not clear and needs deeper investigation. We hypothesize that such patients might have been lost after referral from the point of entry to the point of HIV testing. We also think that poor documentation might be another reason for this finding. This study has several programmatic implications. First, although Zimbabwe has done well in improving the uptake of HIV testing up to 86%, attempts should be made to get it as close to 100% as possible in order to achieve the UNAIDS 90-90-90 targets for ending the AIDS epidemic. As per the recent ZIMPHIA report, Zimbabwe is lagging behind in the first 90 with only 72.9% of estimated PLHIV knowing their HIV status [28]. Second, poor ART uptake among the HIV-positive non-TB patients indicates continued neglect of this priority group and is a matter of grave concern requiring urgent attention. Besides the fact that ART substantially reduces mortality among HIV-positive patients irrespective of TB status [29], it is important to put this group under ART care and follow up as soon as possible for two additional reasons: i) being under regular ART care will facilitate early diagnosis of TB, ii) being eligible for Isoniazid Preventive Therapy (IPT) administration will prevent the development of TB [30]. We suggest the introduction of an indicator in the routine programme to closely monitor HIV status ascertainment and ART and IPT uptake among presumptive TB patients without TB. Third, incomplete documentation was reported, similar to another study in Zimbabwe, thereby stressing the need for improved recording and reporting [16]. Standard Operating Procedures (SORT IT) for data recording and checking registers for data quality during routine supervisory visits might help. Fourth, future research should explore the reasons for poor ART uptake, more among the non-TB group through an in-depth qualitative inquiry involving all relevant stakeholders.

## Conclusion

The study found encouragingly high rates of HIV status ascertainment among presumptive TB patients. However, uptake of ART was poor among those without TB. The national TB and HIV programmes should step up the monitoring of HIV status and ART uptake among presumptive TB patients.

### What is known about this topic

- HIV testing is routinely offered not only to TB patients but also to “presumptive TB patients without TB” but HIV care among the latter is not routinely reported and monitored;
- A study carried out in a single health facility in Zimbabwe in 2009-13 reported high HIV ascertainment and low ART uptake (38%) among presumptive TB patients.

### What this study adds

- HIV status ascertainment continues to be high among presumptive TB patients (86%), though marginally better among TB patients (98%) compared to non-TB (83%);
- Even after implementation of “test and treat” approach, ART uptake (63%) remains poor: worse among patients without TB (57%) compared to those with TB (78%);
- Compared to previous study, ART uptake has improved from 38% to 57%, though still far away from the globally recommended 90-90-90 target.

## Competing interests

The authors declare no competing interests.
